# Anticancer Drugs from Marine Flora: An Overview

**DOI:** 10.1155/2010/214186

**Published:** 2011-02-27

**Authors:** N. Sithranga Boopathy, K. Kathiresan

**Affiliations:** Center of Advanced Study in Marine Biology, Faculty of Marine Sciences, Annamalai University, Parangipettai 608 502, Tamil Nadu, India

## Abstract

Marine floras, such as bacteria, actinobacteria, cyanobacteria, fungi, microalgae, seaweeds, mangroves, and other halophytes are extremely important oceanic resources, constituting over 90% of the oceanic biomass. They are taxonomically diverse, largely productive, biologically active, and chemically unique offering a great scope for discovery of new anticancer drugs. The marine floras are rich in medicinally potent chemicals predominantly belonging to polyphenols and sulphated polysaccharides. The chemicals have displayed an array of pharmacological properties especially antioxidant, immunostimulatory, and antitumour activities. The phytochemicals possibly activate macrophages, induce apoptosis, and prevent oxidative damage of DNA, thereby controlling carcinogenesis. In spite of vast resources enriched with chemicals, the marine floras are largely unexplored for anticancer lead compounds. Hence, this paper reviews the works so far conducted on this aspect with a view to provide a baseline information for promoting the marine flora-based anticancer research in the present context of increasing cancer incidence, deprived of the cheaper, safer, and potent medicines to challenge the dreadful human disease.

## 1. Introduction

Cancer is a dreadful human disease, increasing with changing life style, nutrition, and global warming. Cancer treatments do not have potent medicine as the currently available drugs are causing side effects in some instances. In this context, the natural products derived from medicinal plants have gained significance in the treatment of cancer. According to the WHO, 80% of the world's population primarily those of developing countries rely on plant-derived medicines for the health care [1]. Natural products and their derivatives represent more than 50% of all the drugs in clinical use of the world. Higher plants contribute not less than 25% of the total. Almost 60% of drugs approved for cancer treatment are of natural origin. Fruits and vegetables are the principal sources of vitamins C, B, E, carotenoids, and fibers, and these contribute to the apparent cancer-protective effects of the foods. There is a positive correlation between the increased dietary intake of natural antioxidants and the reduced coronary heart diseases, cancer mortality, as well as longer life expectancy [2, 3]. Herbal drug formulations for the prevention and treatment of cancer appeared in the last three decades, and the interest on natural sources of potential chemotherapeutic agents is continuing.

Antioxidants play an important role in the later stages of cancer development. There is increasing evidence that oxidative processes promote carcinogenesis, although the mechanisms for this are not well understood. The antioxidants may be able to cause the regression of premalignant lesions and inhibit their development into cancer. Preliminary studies have indicated that some antioxidants, particularly *β*-carotene, may be of benefit in the treatment of precancerous conditions such as oral leukoplakia, possibly a precursor of oral cancer [4]. Several herbs and spices including rosemary, sage, thyme, nutmeg, turmeric, white pepper, chilli, pepper, ginger, and plenty of other medicinal plants are reportedly exhibiting antioxidant activity [5–7]. Majority of the active antioxidant compounds are flavonoids, isoflavones, flavones, anthocyanins, coumarins, lignans, catechins, and isocatechins. In addition to these, vitamins C and E, *β*-carotene, and *α*-tocopherol present in natural foods, are known to possess antioxidant potential [8–10]. Thus, potential antioxidant and anticancer properties of plant extracts or isolated products of plant origin can possibly be explored for developing the anticancer drugs [11].

From the past few decades, there has been an upsurge in the search for new plant-derived drugs. This process has facilitated to produce remarkably a diverse array of over 1,39,000 natural products, containing medicinally useful terpenoid derivatives, alkaloids, glycosides, polyphenolics, steroids, and so forth. The National Cancer Institute (NCI) of the United States of America (USA) has screened about 1,14,000 extracts from an estimated 35,000 plant samples against a number of tumor systems [12]. Of the 92 anti-cancer drugs commercially available prior to 1983 in the USA and approved world-wide between 1983 and 1994, approximately 62% can be related to natural origin [13]. Some examples include vinblastine and vincristine (*Catharanthus roseus*), epipodophyllotoxin, an isomer of podophyllotoxin (*Podophyllum peltatum *roots), paclitaxel (*Taxus baccata*, *T. brevifolia, T. canadensis*), camptothecin (*Camptotheca acuminata*), homoharringtonine (*Cephalotaxus harringtonia *var. *drupacea*), elliptinium (*Bleekeria vitensis*), flavopiridol (*Dysoxylum binectariferum*), and ipomeanol (*Ipomoea batatas*). The two plant-derived natural products, paclitaxel and camptothecin were estimated to account for nearly one-third of the global anticancer market, respectively to the tune of about $3 and $9 billion, in the year 2002 [14]. 

Numerous types of bioactive compounds have been isolated from plant sources. Several of them are currently in clinical trials or preclinical trials or undergoing further investigation. Although marine compounds are under-represented in current pharmacopoeia, it is anticipated that the marine environment will become an invaluable source of novel compounds in the future, as it represents 95% of the biosphere [15]. However, development of marine floral compounds as therapeutic agents is still in its embryonic stage due to lack of an analogous ethno-medical history as compared to terrestrial habitats, together with the relative technical difficulties in collecting the marine floral samples. Over the last few decades, significant efforts have been made, by both pharmaceutical companies and academic institutions, to isolate and identify new marine-derived, natural products especially from faunal species. However, the marine floras are only little unexplored and these works are reviewed here as a baseline data for promoting further research in this field.

## 2. Uniqueness of Marine Floral Drugs

Marine floras include microflora (bacteria, actinobacteria, cyanobacteria and fungi), microalgae, macroalgae (seaweeds), and flowering plants (mangroves and other halophytes). Occupying almost 71% of globe, the ocean is rich in biodiversity, and the microflora and microalgae alone constitute more than 90% of oceanic biomass [16]. This vast marine floral resource will offer a great scope for discovery of new drugs. It is increasingly recognized that ocean contains a huge number of natural products and novel chemical entities with unique biological activities that may be useful in finding the potential drugs with greater efficacy and specificity for the treatment of human diseases [17]. It cannot be denied that with 3.5 billion years of existence on earth and experience in biosynthesis, the marine microfloras remain nature's best source of chemicals. The marine organisms produce novel chemicals to withstand extreme variations in pressure, salinity, temperature, and so forth, prevailing in their environment, and the chemicals produced are unique in diversity, structural, and functional features [18]. 

The efforts to extract drugs from the sea started in the late 1960s. However, the systematic investigation began in the mid-1970s. During the decade from 1977 to 1987, about 2500 new metabolites were reported from a variety of marine organisms. These studies have clearly demonstrated that the marine environment is an excellent source of novel chemicals, not found in terrestrial sources. So far, more than 10,000 compounds have been isolated from marine organisms with hundreds of new compounds are still being discovered every year. About 300 patents on bioactive marine natural products were issued between 1969 and 1999 [18]. Some marine organisms are proved to be the potent sources of drugs. These are mostly invertebrates that include sponges, soft corals, sea fans, sea hares, nudibranchs, bryozoans, and tunicates. It is now believed that microbial floras present in the invertebrates are responsible for the production of medicinal compounds. The search is mostly confined to marine faunal species, and floral species are largely ignored. Some of the compounds derived from marine organisms have antioxidant property and anticancer activities, but they are largely unexplored. 

Marine floras have been used for medicinal purposes in India, China, the Near East and Europe, since ancient times. The people of China and Japan have been using seaweeds for consumption. The seaweeds especially brown seaweeds are rich in iodine and hence there is a least incidence of goiter and glandular diseases. History reveals that maritime countries have been using seaweeds as vermifuge, anesthetics and ointment as well as for the treatment of cough, wounds, gout, goiter, venereal disease, and so forth. Sterols and related compounds present in seaweeds have ability to lower blood plasma cholesterol level. Seaweed dietary fibers perform varied range of functions such as antioxidant, antimutagenic, anticoagulant, and antitumor. The seaweeds also play an important role in modification of lipid metabolism in the human body. High intake of calcium, potassium, and sodium is associated with lower mean systolic pressure and lower risk of hypertension. All seaweeds offer an extraordinary level of potassium that is very similar to our natural plasma level. Seaweed extract is interestingly similar to human blood plasma. Two Japanese surgeons have used a novel technique of mixing seaweed compounds with water to substitute whole blood in transfusion and this has been successfully tried in over 100 operations [4].

Although, the use of seaweeds in medicine is not as wide spread as once it was, the use of seaweed polymer extract in pharmacy, medicine, and biochemistry is well established. Clinical trials are also in progress to make diabetic patients free from injection by introducing insulin secreting “jelly capsule” made of seaweed-derived alginic acid [19]. The capsule renders protection to white blood cells and the patient's immune system. Seaweed gums like carrageenan (extracted from red seaweed) or algin (from brown seaweed) are rich sources of soluble fibers [4].

## 3. Anticancer Agents from Marine Floras

### 3.1. Bacteria

Marine microorganisms are a source of new genes, and exploitation of which is likely to lead to the discovery of new drugs and targets. Secondary metabolites produced by marine bacteria have yielded pharmaceutical products such as novel anti-inflammatory agents (e.g., pseudopterosins, topsentins, scytonemin, and manoalide), anticancer agents (e.g., bryostatins, discodermolide, eleutherobin, and sarcodictyin), and antibiotics (e.g., marinone). The contribution of probiotic bacteria, such as lactobacilli and bifidobacteria, is mainly in the control of pathogenic microbes, through production of antibacterial protein namely, bacteriocin [20, 21] and anticancer substances [22]. The dietary supplements of lactobacilli are reportedly decreasing the induction of experimental colon cancer [23]. They stimulate and modulate the mucosal immune system by reducing the production of proinflammatory cytokines through actions on NF*κ*B pathways, increasing production of anti-inflammatory cytokines such as IL-10 and host defense peptides such as *β*-defensin 2, enhancing IgA defenses and influencing dendritic cell maturation as well as modulation of cell proliferation and apoptosis through cell responses to short chain fatty acids [24]. 

Most of the marine animal phyla produce toxins and some studies show that these marine toxins may be produced by marine bacteria associated the animals [25–27]. The microbial toxins are useful in neurophysiological and neuropharmacological studies. For example, bacteria present in *Noctiluca scintillans* are responsible for causing red tides. The major metabolite, macrolactin-A, inhibits B16-F10 murine melanoma cancer cells, mammalian herpes simplex virus (HSV) (types I and II), and protects T lymphocytes against human immunodeficiency virus (HIV) replication [28].

Kahalalide F (KF) is a depsipeptide isolated from the mollusk *Elysia rubefescens *from Hawaii and the compound is believed to be synthesized by microbes associated with the animal. KF induces cytotoxicity and blocks the cell cycle in G1 phase in a p53-independent manner. *In vitro*, KF displays activity against solid tumors with an interesting pattern of selectivity in prostate cancer cell lines. In addition, extensive *in vivo *work demonstrates that the agent has activity in breast and colon cancers.

Only a few marine bacteria can be isolated under laboratory conditions and there is an urgent need to develop new culture techniques to isolate slow-growing bacteria and also to isolate the bacteria that are unique in production of novel natural products [29].

### 3.2. Actinomycetes

For more than 50 years, the soil-derived actinomycetes of terrestrial origin have provided a major pharmaceutical resource for the discovery of antibiotics and related bioactive compounds. However, marine actinomycetes received only very recent attention. Gutingimycin is a highly polar trioxacarcin derivative from *Streptomyces *species, isolated from sediment of the Laguna de Terminos, Gulf of Mexico [30]. The same *Streptomyces *species also yields trioxacarcins D–F, in addition to the known trioxacarcins A–C [30]. Among the antibiotic-producing microbes, marine actinomycetes within the family Micromonosporaceae are very promising. These microbes are found to be a potent sources of anticancer agents that target proteasome function and their industrial potential is validated by several pharmaceuticals.

Thiocoraline is a novel bioactive depsipeptide isolated from *Micromonospora marina*, a marine microorganism located in the Mozambique Strait that inhibits RNA synthesis. The bioactive compound is also selectively cytotoxic against lung and colon cancer cell lines as well as melanoma. Interestingly, the compound exerts preferential antiproliferative effects in colon cancer cell lines with defective p53 systems [31]. Thiocoraline represents a model of an anticancer agent acquired from marine microorganisms and illustrates how the problems of drug supply can be overcome by artificial culture. 

### 3.3. Marine Fungi

A rich profile of biologically active metabolites is described from filamentous fungi of terrestrial origin, especially from just three genera: *Penicillium, Aspergillus, *and* Fusarium* [32]. However, the marine fungi are least studied than terrestrial counterparts and other ecological groups. Obligate marine fungi are still an unexplored resource, although, marine facultative fungi, have been studied due to their production of new metabolites which are not found in terrestrial fungi. Recently more interest has been generated on studying biologically active metabolites from higher fungi (Basidiomycetes), endophytic fungi and filamentous fungi from marine habitats, the symbiotic lichens. 

In one study, the lignicolous fungus *Leptosphaeria oraemaris *(Pleosporaceae) yielded leptosphaerin [33, 34]. A further study of the same fungal species yielded none of the previously found metabolites, but the polyketides, leptosphaerolide, its *o*-dihydroquinone derivative, and leptosphaerodione [35]. This leads to a conclusion that the production of secondary metabolites might be highly dependent on the culture conditions and the origin of the strains. To produce these metabolites and to maximize the potential chemical diversity, they need to be grown in various nutrient-limited media. For example, media for *Penicillium *spp. that are deficient in carbon can produce penicillins, those that are phosphorus-limited can produce cephalosphorins and vancomycin, and those that are nitrogen-limited can produce carbapenems [36].

Marine-derived fungi are known to be a source of antioxidative natural products: (i) Acremonin A from* Acremonium* sp. [37] and (ii) Xanthone derivative from *Wardomyces anomalus* [38]. Reactions of free radicals, such as super-oxide radical, hydroxyl radical, peroxyl radical and other reactive oxygen and nitrogen are associated with diseases such as atherosclerosis, dementia, and cancer. Antioxidants delay or prevent oxidative damage and thus they may be useful as therapeutics or food additives. 

### 3.4. Micro Algae

Marine blue-green algae (Cyanobacteria) are considered to be one of the potential organisms which can be the richest sources of known and novel bioactive compounds including toxins with potential for pharmaceutical applications [39, 40]. Some of the marine cyanobacteria appear to be potential sources for large-scale production of vitamins (B complex, E) of commercial interest. Scytonemin is a protein serine/threonine kinase inhibitor [41], isolated from the cyanobacterium *Stigonema *sp. and this compound is a yellow-green ultraviolet sunscreen pigment, known to be present in the extracellular sheaths of different genera of aquatic and terrestrial blue-green algae. Scytonemin regulates mitotic spindle formation as well as enzyme kinases involved in cell cycle control and the compound also inhibits proliferation of human fibroblasts and endothelial cells. Thus scytonemin may provide an excellent drug as protein kinase inhibitors to have antiproliferative and anti-inflammatory activities [42]. 

More than 50% of the marine cyanobacteria are potentially exploitable for extracting bioactive substances which are effective in either killing the cancer cells by inducing apoptotic death, or affecting the cell signaling through activation of the members of protein kinase-c family of signaling enzymes. The cell extracts of *Calothrix* isolates inhibit the growth *in vitro* of a chloroquine-resistant strain of the malarial parasite, *Plasmodium falciparum,* and of human HeLa cancer cells in a dose-dependent manner [43]. Bioassay directed fractions of the extracts have led to their isolation and structural characterization of Calothrixin A (I) and B (II), pentacyclic metabolites with an indole [3, 2 – j] phenanthridine alkaloids which exert their growth inhibitory effects at nanomolar concentrations [43]. Another compound, Curacin-A, isolated from the organic extracts of Curacao collections of *Lyngbya majuscula* is an exceptionally potent antiproliferative agent as it inhibits the polymerization of the tubulin and it also displays the inhibitory activity selectively on colon, renal, and breast cancer-derived cell lines [28]. 

Largazole is unique chemical scaffold with impressive antiproliferative activity derived from *Symploca* sp. [44]. The apratoxins are another class of cyanobacterial compounds that inhibit a variety of cancer cell lines at nanomolar concentrations. The parental compound, apratoxin A, isolated from a strain of *Lyngbya boulloni *shows cytotoxicity to an adenocarcinoma [45]. The coibamide A is a compound derived from a strain of *Leptolyngbya* [46], and it exhibits significant cytotoxicity against NCIH460 lung and mouse neuro-2a cells. The cytotoxicity is a common mechanism of action for many cyanobacterial compounds [47]. 

In recent times, the most significant discoveries are of borophycin, cryptophycin 1 & 8, and cyanovirin. Borophycin is a boron-containing metabolite, isolated from marine cyanobacterial strains of *Nostoc linckia *and *N. spongiaeforme *var. *tenue *[48]. The compound exhibits potent cytotoxicity against human epidermoid carcinoma (LoVo) and human colorectal adenocarcinoma (KB) cell lines [49]. Borophycin is related both to the boron containing boromycins isolated from a terrestrial strain of *Streptomyces antibioticus *and to the aplasmomycins isolated from a marine strain of *Strepetomyces griseus *(actinomycetes) [48]. 

Cryptophycin 1 was first isolated from *Nostoc* sp. ATCC 53789 by researchers at Merck and found to be a potent fungicide. As it was highly toxic, it was disregarded as a natural product lead. Subsequently, the same compound isolated from *Nostoc* sp. GSV 224 exhibited potent cytotoxicity against human tumor cell lines and good activity against a broad spectrum of drug sensitive and drug-resistant murine and human solid tumors [50]. Nevertheless, cryptophycin 1 again appears to be too toxic to become a clinical candidate. This leads to a detailed structure-function study which has resulted in the isolation of cryptophycin 8, a semisynthetic analogue with greater therapeutic efficiency and lower toxicity than cryptophycin 14 *in vivo* [51]. Although neither cryptophycin, nor any of its analogues have entered clinical trails to-date, but interest in these compounds continues.

### 3.5. Macro Algae (Seaweed)

Seaweeds are important sources of protein, iodine, vitamins, and minerals and hence, their metabolites have shown promising activities against cancer incidences [52]. The seaweeds also contain high amounts of polyphenols such as catechin, epicatechin, epigallocatechin gallate, and gallic acid, as reported in *Halimeda *sp. (Chlorophyceae) [53]. In the past three decades, many researchers have worked on the antioxidant, antitumor, and immunomodulating activities of seaweeds [54]. Edible seaweed like *Palmaria palmate *is shown to be effective antioxidant, capable of inhibiting cancer cell proliferation [55]. The alcoholic extract of the red alga* Acanthophora spicifera *exhibits tumoricidal activity on Ehrlich's ascites carcinoma cells developed in mice at a dose of 20 mg/kg, comparable to the standard drug, 5-flurouracil. This is evidenced by increase in the mean survival time, decrease in tumor volume, and viable cell count. The smear study exhibits membrane blebbing, vacuole formation, and reduction in staining intensity, which further ascertains the tumoricidal activity. The seaweeds *Acanthaphora spicifera, Ulva reticulata, Gracilaria foliifera, and Padina boergesenii *of the Gulf of Mannar region are reportedly exhibiting cytotoxic activity in their alcoholic extracts [56, 57]. 

Algae have gained special interest owing to their biological properties. There are many reports on the immunomodulating and antitumor activities of algae [54, 58–71]. An extract from the brown seaweed *Sargassum thunbergii *has shown antitumour activity [72] and inhibition of tumour metastasis in the rat mammary adeno carcinoma cell (13762 MAT) [73]. Moreover, low-molecular weight fucoidan isolated from *Ascophyllum nodosum *shows an anti-proliferative effect on both normal and malignant cells, including fibroblasts (Hamster Kidney Fibroblast CCL39), sigmoid colon adenocarcinoma cells (COLO320 DM), and smooth muscle cells [74]. Fucoidans exhibit antitumour, anticancer, antimetastatic, and fibrinolytic properties in mice [73, 75]. Stylopoldione, isolated from *Stypodium *sp. is a potent cytotoxic metabolite, which halts mitotic spindle formation [76]. The compound Condriamide-A from *Chondria *sp. exhibits cytotoxicity towards human nasopharyngeal and colorectal cancer cells [77]. Caulerpenyne from *Caulerpa *sp. shows its bioactivity against human cell lines and to have anticancer, antitumour, and antiproliferating properties. Two compounds, meroterpenes and usneoidone, showing antitumour properties have been isolated from *Cystophora* sp. [78–81] Phloroglucinol and its polymers, namely, eckol (a trimer), phlorofucofuroeckol A (a pentamer), dieckol, and 8,8′-bieckol (hexamers) isolated from the brown alga *Eisenia bicyclis* are shown to have antioxidant activity [82, 83]. 

The brown alga *Eclonia cava* has been hydrolyzed by using five different types of carbohydrases such as AMG, Celluclast, Termamyl, Ultraflo, and Viscozyme to produce enzymatic extracts and proved them to be potential natural water-soluble antioxidants with dose dependent radical scavenging activities [94]. Further studies have shown that a sulfated polysaccharide purified from the same algal species selectively and dose-dependently suppresses the proliferation of the cancer cell lines *in vitro *[95]. The polysaccharide is composed of fucose (82%), galactose (14%), and small amounts of xylose and mannose. Its high anticoagulant activity has also been investigated for its antiproliferative effect on murine colon carcinoma (CT-26), human leukemic monocyte lymphoma (U-937), human promyelocytic leukemia (HL-60), and mouse melanoma (B-16) cell lines. The growth inhibition rate of CT-26 cells increases consistently with the sample concentration, in which the highest activity (around 40%) is recorded at 100 *μ*g mL^−1^ sample [95]. The apoptosis induction is confirmed by the cell cycle analysis, while pronounced sub-G1 phase arrests of 9.5% and 13.8% are also clearly observed when the cells are treated at 15 and 30 *μ*g mL^−1^ of the sulphated polysaccharides in the U-937 cell line. The compound dose dependently enhances the DNA fragmentation on the U-937 cell line as observed after 24-h incubation. The western blot analyses conducted with several antibodies such as caspase-7, caspase-8, Bax, Bcl-xL, and PARP and ECSP have exhibited a clear effect on the caspase-7 and -8 which cleave protein substrates, including PARP, an inducer of apoptosis responsible for DNA cleavage [95].

### 3.6. Mangroves and Other Higher Plants

Mangroves have long been used in fisher-folk medicine to treat diseases [96, 97]. Sixteen plants are the possible source of anticancer drugs, based on traditional knowledge and preliminary scientific work (Table 1). A sulphur containing alkaloid, 1,2-dithiolane (Brugine) isolated form *Bruguiera sexangula* displays antitumor activity against Sarcoma 180 and Lewis. Tannin from the same plant also exhibits anticancer activity against lung carcinoma. A ribose derivative of 2-Benzoxazoline isolated from *Acanthus ilicifolius* shows anticancer and antiviral activities [98]. Tea from the mangrove plant *Ceriops decandra* is shown to successfully prevent the dimethyl benz[a]anthracine-induced hamster buccal pouch carcinogenesis; consequently it enhances beneficial bacteria like lactobacilli in oral cavity of the animals [88]. 

## 4. Chemical Constituents of Marine Flora

Marine floras are rich in biologically active and medicinally potent chemicals. Polyphenols and polysaccharides are the most predominant group of compounds which are applicable for antioxidant and anticancer activities. There are more than 40,000 different species of phytoplankton, 680 species of marine algae belonging to Rhodophyta, Phaeophyta, Chlorophyta commonly known as red, brown, and green seaweeds, respectively, and 71 mangrove plant species have been documented in the global marine biotope. They provide essential fatty acids, ionic trace minerals, vitamins, enzymes, bioflavonoids, amino acids, and other nutrients. 

### 4.1. Polyphenols

Polyphenols are widely distributed in plants and they are reportedly acting as free radical scavengers, antimicrobial and anticancer agents [99, 100]. Marine plants such as seaweeds, sea grass, and mangroves also contain high amounts of polyphenols such as phenolic acids, flavonoids, anthocyanidins, lignin, tannins, catechin, epicatechin, epigallocatechin, and gallic acid [53, 101]. These polyphenolic compounds have shown many health-benefiting bioactivities, such as antioxidant, anticancer, antiviral, anti-inflammatory, and an ability to inhibit human platelet aggregation [102–104]. Some studies have shown a positive correlation between the increased dietary intake of natural antioxidants and the reduced coronary heart disease, cancer mortality, as well as longer life expectancy [2, 3]. Moreover, they are natural metal chelators with high antioxidant activity that may be successfully used to prevent a variety of toxic metal ion-induced organ dysfunctions [105]. Earlier reports suggest that polyphenols may regenerate *α*-tocopherol through reduction of the *α*-tocopheroxyl radical [106]. A close association between anticarcinogenic activity and antioxidant activity has been reported in a chemically induced mouse carcinoma system with low-molecular weight polyphenols [107–110].

**Figure 1:**
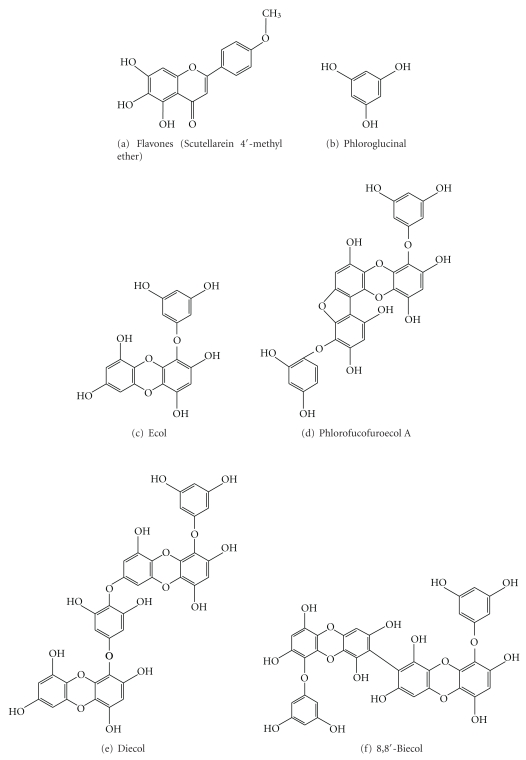
Anticancer polyphenolic compounds from marine floras.

The marine red algae like *Osmundea pinnatifida *has been documented for its antimicrobial, antifungal, anti-leishmanial, and antioxidant [111–114] activities. Scutellarein 4′-methyl ether (Figure 1(a)) has antiallergic [115], anticancer and anticytotoxic activities *in vitro *and *in vivo* [116].

**Figure 2:**
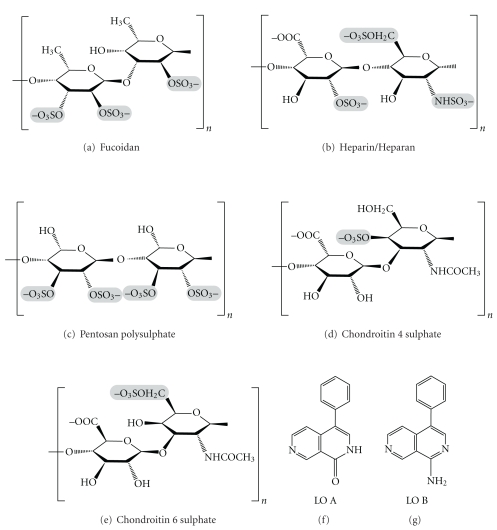
Anticancer polysaccharides from marine floras.

Terrestrial and marine polyphenols are similar in some respects, but different fundamentally in their chemical structures. Terrestrial polyphenols are polymers based on flavonoids or gallic acids. Marine algal polyphenols, phlorotannins, which are only known in brown algae, are restricted to polymers of phloroglucinol (1,3,5-trihydroxybenzene) [117]. Six phlorotannins have been detected by HPLC analysis in the brown seaweeds, *Eisenia bicyclis* and *Eclonia kurome,* and they are phloroglucinol (0.7%), an unknown phloroglucinol tetramer (MW 478, 3.4%), eckol (7.5%), phlorofucofuroeckol A (21.6%), dieckol (21.9%), 8,8′-bieckol (24.0%), and other unknown compounds (20.9%), in *E. bicyclis, * and these compounds are also present in *E. kurome,* respectively, at concentrations of 2.2, 0.6, 8.5, 27.6, 23.6, 6.8, and 31.7% (Figures 2(b), 2(c), 2(d), 2(e), and 2(f)). The crude phlorotannins extracted from brown algae have inhibitory effects on HAase [118]. The half maximal inhibition (IC_50_) values of crude phlorotannins of *E. bicyclis* and *E. kurome*, two terrestrial polyphenols (catechin, EGCG), inhibit four times stronger than that by an anti-allergic drug (DSCG) [119]. 

Edible seaweeds contain a range of potentially bioactive components including polyphenols and phlorotannins [120–123]. Edible seaweed like *Palmaria palmate* is shown to be an effective antioxidant, capable of inhibiting cancer cell proliferation [55]. The enzymatic hydrolysis of the brown seaweed *Ecklonia cava* yields high amount of compounds with enhanced biological activities as compared with water and organic extract counterparts [94]. Phloroglucinol and its polymers, namely, eckol (a trimer), phlorofucofuroeckol A (a pentamer), dieckol, and 8,8′-bieckol (hexamers) isolated from *Eisenia bicyclis*, have a potential antioxidant activity [82]. The phlorotannins isolated from *Ecklonia kurome *act as antiplasmin inhibitor; however, other bioactivities of phlorotannins, from a human physiological viewpoint, are still obscure [124]. 

Polyphenolic compounds inhibit cancer cells by xenobiotic metabolizing enzymes that alter metabolic activation of potential carcinogens, while some flavonoids can also alter hormone production and inhibit aromatase to prevent the development of cancer cells [125]. The mechanism of action of anticancer activity of phenolics is by disturbing the cellular division during mitosis at the telophase stage. Phenolics reduce the amount of cellular protein and mitotic index, and the colony formation during cell proliferation of cancer cells [126]. Several studies exhibit a close relationship between antioxidant activities and total phenolic content [127–129].

Use of phytosubstances to improve or enhance their effects with safety in foods is significantly focused in daily food. The activities of diverse constituents vary in their ability by quenching effects against active free radical oxygen by carotenes and cryptoxanthins, and polyphenols and flavonoids, by inhibition of absorption into small intestine by dietary fibres, or by regulation on efflux and influx of ions in cell membranes by minerals to inhibit tumors [130–132]. 

The uses of saponins are natural detergents, well known to primitive people as fish poisons. The interesting pharmacological properties associated with the Chinese drug “giwieng” are considered a panacea and other interesting biological activities such as spermicidal [133], molluscicidal [134], antimicrobial, anti-inflammatory, and cytotoxic activities [135]. *Avicennia officinalis* produces pharmacologically significant steroidal saponins, sapogenisis, and sapogenins. Liomonds (modified terpenes) have attracted much attention recently because of their remarkable insect antifeedent and growth-regulating activities [136]. There are many types of flavonoids such as flavones, catechins, chalcones, flavanols and isoflavonoids which exhibit antioxidant activity towards a variety of oxidizable compounds [137].

### 4.2. Polysaccharides

Over the last few years, medical and pharmaceutical industries have shown an increased interest in seaweed-derived polysaccharides. Polysaccharides or glycans are a group of major chemical compounds with the most common constituents of monosaccharide like D-glucose, but D-fructose, D-galactose, L-galactose, D-mannose, L-arabinose, and D-xylose are also frequently present. Some monosaccharide derivatives found in polysaccharides include the amino sugars (D-glucosamine and D-galactosamine) as well as their derivatives (*N*-acetylneuraminic acid and *N*-acetylmuramic acid) and simple sugar acids (glucuronic and iduronic acids). Polysaccharides of algal origin include alginates, agar, and carrageenans. Agar is an unbranched polysaccharide present in the cell membranes of red algae, primarily from the genera *Gelidium *and *Gracilaria,* and it is the primary structural support for the algal cell walls. Chemically, it is constituted by galactose sugar molecules. Carrageenans are polysaccharides of galactan with alternating 1,3- and 1,4-linked galactose residues, which fill spaces between the cellulosic plant structure of seaweeds. 

The active components contained in algal polysaccharides are mainly sulfated ones [63–67, 69, 70]. Most studies support that sulfated polysaccharides can enhance the innate immune response by promoting the tumoricidal activities of macrophages and natural killer cells [138–141]. Antigen-presenting cells migrate into and out of tumour tissue to present tumour antigen to T-helper cells, as well as to produce cytokines, such as interleukin-1 beta and TNF-alpha that stimulate T-helper cells. As a result, T-helper cells promote the activity of cytotoxic T-cell, which has the strong cytotoxic effect on tumour cells. Sulfated polysaccharides can enhance the adaptive immune response by promoting such process [140, 142–144]. Recent studies have implicated that sulfated polysaccharides recognize a range of cell adhesion systems. Sulfated polysaccharide can bind to CD2, CD3, and CD4 in T lymphocytes and enhance the proliferative response of T lymphocytes [145–147]. B-1, a sulfated polysaccharide isolated from the culture filtrate of marine *Pseudomonas *sp., induces apoptosis of human leukaemic cells (U937) [148]. PI-88, a sulfated oligosaccharide, induces apoptosis of pancreatic islet carcinoma [149]. Internalized sulfated glycosaminoglycans interfere with transcription function and subsequently induce apoptosis of murine melanoma cells [150]. 

Fucoidan is one of the representative sulfated polysaccharides (sulphated L-fucose) derived from cell wall of brown algae [65, 66, 151]. Fucoidan-induced apoptosis in human lymphoma HS-Sultan cell lines is accompanied by the activation of caspase-3 and down-regulation of extracellular signal-regulated kinase pathway [152]. Fucoidans have diverse biological properties, ranging from relatively simple mechanical support functions to more intricate effects on cellular processes [151] and binding proteins such as adhesion proteins [153], growth factors [154], cytokines [155], and a variety of enzymes, including coagulation proteases [156]. As a result, they can participate like glycosaminoglycans (GAGs) in cell adhesion, migration, proliferation, and differentiation. They can also modulate clinically relevant phenomena such as angiogenesis, tumor metastasis, and atherosclerosis [157]. For the past decade, fucoidans isolated from different species have been extensively studied due to their varied biological activities, including anticoagulant, antithrombotic, antivirus, antitumor, immunomodulatory, anti-inflammatory, blood lipids reducing, antioxidant, and anticomplementary activities against hepatopathy, uropathy and renalpathy, gastric protective effects, and therapeutic potential in surgery. Compared with other sulfated polysaccharides, fucoidans have been increasingly investigated in recent years to develop the drugs or functional foods [158]. The type of fucoidan, its sulphation and molecular weight, and the conformation of its sugar residues vary with the seaweed species [151, 159]. 

Sulphation is critical for fucoidan activity *in vivo*. In particular, desulphated fucoidan fails to promote angiogenesis *in vitro *[160] or to induce immature CD34+ cell mobilization *in vivo *[161]. Native fucoidan-induced mobilization is abolished in the presence of protamine [162]. The predominant sulphation pattern consists of a trisulphated disaccharide repeat similar to that found in heparin [163, 164]. Yet heparin has no effect on angiogenesis induced by HUVEC *in vitro *[165] and does not induce significant immature CD34+ cell mobilization [161]. Furthermore, heparan sulphate (Figure 2(b)), pentosan sulphate (Figure 2(c)), and chondroitin sulphate (Figures 2(d) and 2(e)), which exhibit anticoagulant activities, inhibit angiogenesis *in vitro. *Fucoidan can disrupt heparan sulphate-growth factor/cytokine complexes and can substitute for cell-surface heparan sulphates in stabilizing the growth factor/growth factor receptor interaction. Fucoidan may mediate growth factor-induced EPC differentiation by interacting with a “receptor” that promotes endothelial cell adhesion, migration, proliferation and differentiation, and that cooperates with a growth factor receptor, transducing the intracellular signals required to induce the angiogenic phenotype. This putative fucoidan receptor might contain a carbohydrate-binding domain that interacts with the fucoidan carbohydrate backbone [157].

#### 4.2.1. Alkaloids

The term alkaloid was first proposed by Meissner in 1819 to characterize these “alkali-like” compounds found in plants [166], but it was not precisely defined [167]. With time, the definition has changed [168] to a compound that has nitrogen atom(s) in a cyclic ring. Numerous biological amines and halogenated cyclic nitrogen-containing substances are included in the term alkaloid. The latter could not be found in terrestrial plants and is specific from marine organisms including marine algae. Alkaloid chemistry and its anticancer activities have been widely studied in terrestrial plants, but the number of studies in marine plants are insignificant. Morphine was the first alkaloid extracted from a terrestrial plant in 1805 as reported by Kappelmeier [169], and hordenine was the first alkaloid isolated from marine algae in 1969 [170, 171]. Today approximately two thousand alkaloids are known. They occur abundantly in terrestrial plants and rarely in marine algae. 

Among several types of compounds obtained from plants, alkaloids have traditionally been of interest due to their pronounced physiological activities in animals and humans [172]. The most famous examples of anticancer alkaloids are taxol (clinically available since 1994) from the western yew, *Taxus brevifolia*, and camptothecin and derivatives, currently in clinical trials, from *Camptotheca acuminata *[14, 173, 174]. The alkaloid taspine hydrochloride founded in Sangre de Grado plant is also considered a potential anticancer agent [175], and homoharringtonine, an alkaloid isolated from the Chinese tree *Cephalotaxus harringtonia *(Cephalotaxacea), has shown efficacy against various leukemias [176]. The isolation of vinca alkaloids such as vinblastine and vincristine from the Madagascar periwinkle, *Catharanthus roseus *G. Don. (Apocynaceae), has opened a new era of the use of alkaloids as anticancer agents. They were the first agents entered to clinical use for the treatment of cancer [177]. Vinblastine and vincristine are primarily used in combination with other cancer chemotherapeutic drugs for the treatment of a variety of cancers, including leukemias, lymphomas, advanced testicular cancer, breast and lung cancers, and Kaposi's sarcoma [177]. 

The alkaloids found in marine algae may be divided into three groups: Phenylethylamine alkaloids, Indole and halogenated indole alkaloids, and other alkaloids. Structurally, the alkaloids isolated from marine algae mostly belong to the phenylethylamine and indole groups. Biological activities of these alkaloids were not fully investigated. Alkaloids of marine algae are relatively rare, when compared with terrestrial plant alkaloids. Research on marine drugs has largely focused on finding drugs for cancer treatment. There are two derivatives: lophocladine A (Figure 2(f)) and lophocladine B (Figure 2(g)) isolated from a red alga *Lophocladia *sp., collected from Fijian Island, New Zealand [178] and their anticancer activity has been proved successfully in various cancer cell lines [168].

**Figure 3:**
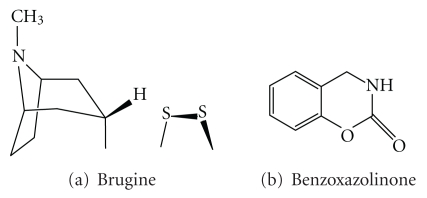
Anticancer alkaloids from marine floras.

Coastal mangroves do contain alkaloids of anticancer activity [98]. “Rhizophrine” is an alkaloid, a major constituent of the leaves of *Rhizophora mucronata* and *R. stylosa.* Similarly the presence of acanthicifolin in *Acanthus illicifolius*, brugine (a sulphur containing alkaloid; Figure 3(a)) in *Bruguiera sexangula,* and benzoquinones (Figure 3(b)) in *Aegiceras corniculatum* and *Kandelia kandel * has been recorded.

## 5. Mechanisms for the Anticancer Activity of Marine Plants

DNA damage is considered to be one of the most important steps leading to cancer. A marker of mutagenic DNA damage will be useful in the estimation of cancer risk of various populations and in monitoring the effects of chemoprevention. Much of this damage is oxidative in nature. It is estimated that a typical human cell experiences about 10.000 oxidative “hits” to its DNA each day. DNA repair enzymes remove most of the damage. Oxidative lesions to DNA accumulate with age and so does the risk of cancer [4].

**Antioxidants**

Several mechanisms are defending against free radicals and other reactive oxygen species (ROS) in human system. Various defenses are complementary to one another because they act on different oxidants or in different cellular compartments. One important line of defense is a system of enzymes, including superoxide dismutase (SOD), glutathione peroxidase (GPx), and catalase as well as several exogenously acquired radical-scavenging substances such as vitamins E and C and carotenoids [179]. Under normal conditions, the high concentrations of SOD maintain superoxide concentrations at a level too low to allow the formation of peroxynitrite. It is also important to mention that the antioxidant reduces glutathione (GSH). GSH is ubiquitous in aerobic tissues, and although it is not a nutrient, it is synthesized from sulfhydryl-containing amino acids and is highly important in intermediary antioxidant metabolism [180]. Nutrition plays a key role in maintaining the body's enzymatic defences against free radicals. Several essential minerals including selenium, copper, manganese, and zinc are involved in the structure or catalytic activity of these enzymes [180].

Unlike other vitamins, vitamin E is not shown to be directly associated with the function of any enzyme system [181]. Its only established role is that of an antioxidant and a scavenger of free radicals, making it effective as a protector of the integrity of lipids and phospholipid membranes. As an antioxidant, vitamin E is strongly interactive with other dietary systemic antioxidants such as vitamin C and glutathione. Accumulating evidence suggests that vitamin E may have several other functions, including modulation of gene expression and inflammatory responses [182].

Vitamin C is a powerful antioxidant because it can donate a hydrogen atom and form a relatively stable ascorbyl free radical (i.e., L-ascorbate anion). As a scavenger of ROS, ascorbate is shown to be effective against the superoxide radical anion, hydrogen peroxide, the hydroxyl radical, and singlet oxygen [183, 184]. Vitamin C also scavenges reactive nitrogen oxide species to prevent nitrosation of target molecules [185]. The ascorbyl free radical can be converted back to reduced ascorbate by accepting another hydrogen atom or it can undergo further oxidation to dehydroascorbate. Dehydroascorbate is unstable but is more fat soluble than ascorbate and is taken up 10–20 times more rapidly by erythrocytes, where it will be reduced back to ascorbate by GSH or NADPH from the hexose monophosphate shunt [186]. Thus, mechanism exists to recycle vitamin C, which is similar to vitamin E.

Free radicals are a product of tissue metabolism, and the potential damage which they can cause is minimized by the antioxidant capacity and repair mechanisms within the cell. Thus in a metabolically active tissue cell in a healthy subject with an adequate dietary intake, damage to tissue will be minimal and most of the damage, if it does occur, will be repaired [187]. Despite the fact that the marine plants possess application in food and in the pharmaceutical industry, the antioxidant and anticancer activities of many types of plants are still unexplored.

**Immunomodulation and Apoptosis**

Apoptosis is a complex process that involves many different signaling pathways and results in a multitude of changes in the dying cells. The apoptotic machinery is triggered as a result of a shift in the balance of anti- and proapoptotic proteins. Up regulation of antiapoptotic proteins, down regulation of proapoptotic proteins, and decreased expression of caspases may lead to decreased apoptosis. Evasion of apoptosis is recognized to facilitate cancer development by blocking differentiation, promoting angiogenesis, and increasing cell motility, invasion, and metastasis [188]. Dysregulation of apoptotic signaling can play a vital role in diseases with insufficient apoptosis leading to cancer.

The proapoptotic member of the Bcl-2 family such as Bim, a BH3 induces apoptosis by binding to and inhibiting the function of antiapoptotic proteins such as Bcl-XL and Bcl-w. In addition, Bim is reportedly inducing cytochrome C release from the mitochondria [189]. The release of cytochrome C from the mitochondria is also induced by caspase 8, an initiator caspase that links the death receptor and mitochondrial pathways of apoptosis. Caspase 3 is an effector caspase that executes cell death by cleavage of proteins, vital for cell survival [190].

Induction of apoptosis is one of the active strategies to arrest proliferation of cancer cells. Radiation and chemical agents like tamoxifen, capable of inducing apoptosis, have been used to treat cancer [191, 192]. Many chemopreventive agents exert their anticarcinogenic effects by inducing apoptosis [193]. The apoptosis inducing effect of plant extracts may be attributed to up regulated immune surveillance, increased macrophage, and activations of death-inducing signal complex. Natural dietary constituents such as curcumin and resveratrol have been reported to induce apoptosis in malignant cells *in vitro* [194]. The marine phytochemicals also can activate the macrophages and induce apoptosis. Fucoidan from *Laminaria japonica *can restore the immune functions of immunosuppressed mice, and it is an immunomodulator acting directly on macrophage and T lymphocyte [195]. It can also promote the recovery of immunologic function in irradiated rats. The mechanism is associated with the arrest of lymphocyte apoptosis by fucoidan [196, 197]. Fucoidan can induce the production of interleukin-1 (IL-1) and interferon-*γ* (IFN-*γ*) *in vitro*. It enhances the functions of T lymphocyte, B cell, macrophage, and natural killer cell (NK cell) and also promotes the primary antibody response to sheep red blood cell (SRBC) *in vivo *[198]. High molecular-weight fucoidan prepared from *Okinawa mozuku *promotes an increase in the proportion of murine cytotoxic T cells [199]. Fucoidan from *Fucus vesiculosus *has immunostimulating and maturing effects on dendritic cells (DCs), which are powerful antigen-presenting cells, via a pathway involving nuclear factor-*κ*B (NF-*κ*B) [200].

**Nutritional Values and Anticancer Effects**

Marine plants play an important role to fulfill the requirement of food and nutrition for rectifying the human ailments. Most diets that are protective against cancer are mainly made up from foods of plant origin. Higher consumption of several plant foods probably protects against cancers. The “plant-based” diets give more emphasis to those plant foods that are high in nutrients, high in dietary fiber (and so in non-starch polysaccharides), and low in energy density. Non-starchy vegetables, and fruits, probably protect against some cancers [201].

Seaweeds are used extensively for human consumption and they contain other interesting components or traditional medicinal value with curative powers for a variety of diseases (tuberculosis, arthritis, colds, influenza, cancer, etc.). Most people unknowingly utilize seaweed products daily in the form of processed food items like processed dairy, meat, and fruit products and domestic commodities like paint, toothpaste, solid air fresheners, cosmetics, and so forth. Seaweeds are excellent source of vitamins A, Bl, B12, C, D & E, riboflavin, niacin, pantothenic acid and folic acid 3, 4 as well as minerals such as Ca, P, Na, K. Their amino acid content is well balanced, containing most of the essential amino acids needed for life and health. They have more than 54 trace elements required for human body's physiological functions in quantities greatly exceeding vegetables and other land plants [202].

## 6. Conclusion

**Figure 4:**
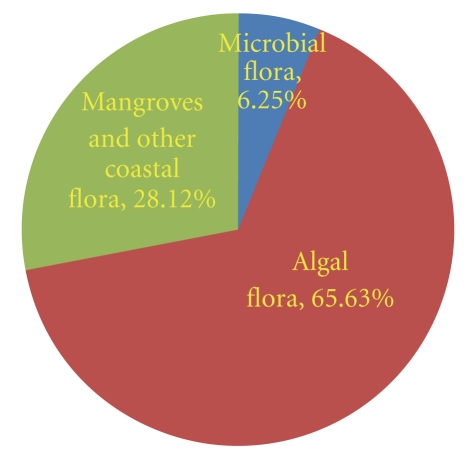
Relative contribution of different marine floral components to anticancer compounds.

Increasing global warming, malnutrition, and various environmental insults continue to increase the incidences of cancer. According to the American Cancer Society, the global burden is expected to grow as 27 million new cancer cases and 17.5 million cancer deaths simply due to the growth and aging of the population by 2050. Natural derivatives play an important role to prevent the cancer incidences as synthetic drug formulations cause various harmful side effects to human beings. Marine floras are potential source of anticancer compounds, but they are least explored (Table 1). Of the anticancer compounds extracted so far, the marine algae contribute 65.63%, the mangroves 28.12%, and the bacteria 6.25%, (Figure 4). Owing to a diverse chemical ecology, the marine organisms especially marine flora have a great promise for providing potent, cheaper, and safer anticancer drugs, which deserve an extensive investigation.

## Figures and Tables

**Table 1:** Some of the marine floral derivatives and their anticancer activities.

Marine flora	Chemical	Biological activity	Reference
Microbial flora			

*Microcystis aeruginosa*	MicroviridinToxin BE-4, Siatoxin	Antibiotic, anticancer	[84, 85]
*Streptomyces peucetius*	Daunorubicin	Anticancer activities on acute myeloid leukemia and acute lymphocytic leukemia	[86]

Algal flora			

Cyanobacteria *Nostoc linckia *and *Nostoc spongiaeforme *var. *tenue *	Borophycin	Cytotoxicity against human epidermoid carcinoma (LoVo) and human colorectal adenocarcinoma activity	[48]
Cyanobacteria	Apratoxins	Inhibit a variety of cancer cell lines	[45]
* Nostoc linckia*	Cyptophycin 1	Cytotoxicity against human tumor cell lines and human solid tumors	[50]
*Nostoc spongiaeforme*	Cryptophycin 8	Greater therapeutic efficiency and lower toxicity than cryptophycin 14 *in vivo *	[51]
*Stylopodium *sp.	Stypoldione	Cytotoxic	[76]
*Chondria *sp.	Condriamide A	Cytotoxicity	[77]
*Caulerpa *sp.	Caulerpenyne	Cytotoxicity, anticancer, antitumour, and antiproliferating activity	[78–80]
*Cystophora *sp.	Meroterpenes and Usneoidone	Antitumour	[81]
*Symploca* sp.	Largazole	Antiproliferative activity	[44]
*Lyngbya boulloni*	apratoxin A	Cytotoxicity to adenocarcinoma	[45]
*Leptolyngbya *sp.	coibamide A	Cytotoxicity against NCIH460 lung and mouse neuro-2a cells	[46]
*Stigonema *sp.	Scytonemin	Antiproliferative and anti-inflammatory activities	[41]
*Acanthophora spicifera*	Crude	Tumoricidal activity on Ehrlich's ascites carcinoma cells developed in mice	[56, 57]
*Acanthophora spicifera*	Crude	Antioxidants and inhibiting cancer cell proliferation	[56, 57]
*Palmaria palmata*	Phloroglucinol and its polymers, namely, eckol (a trimer), phlorofucofuroeckol A (a pentamer), dieckol, and 8,8′-bieckol (hexamers)	Antioxidant activity of the phlorotannins	[55]
*Eisenia bicyclis*	Phloroglucinol and its polymers, namely eckol (a trimer), phlorofucofuroeckol A (a pentamer), dieckol, and 8,8′-bieckol (hexamers)	Antioxidant activity of the phlorotannins	[82, 83]
*Sargassum thunbergii *	Crude	Antitumour activity, inhibition of tumour metastasis in rat mammary adeno carcinoma cell (13762 MAT)	[72, 73]
*Ascophyllum nodosum *	Fucoidan	Antiproliferative antitumour, anticancer, antimetastatic, and fibrinolytic	[74, 75]

Mangroves and other coastal plants			

*Ceriops decandra*	Lignins	Antioxidant	[87]
*Ceriops decandra*	Mangrove tea	Anticancer	[88]
*Acanthus ilicifolius *	Ribose derivatives of benzoxazoline	Anticancer	[89, 90]
*Calophyllum inophyllum *	Xanthone, biflavonoids, benzophenones, neoflavanoids, and coumarin derivatives	Anticancer, antitumour, and lipid peroxidation	[91, 92]
*Excoecaria agallocha *	Diterpenes exhibited remarkable antitumour promoting activity *in vivo* on two-stage carcinogenesis test of tumour	Antitumour activity of methanolic extract based on three assays: (i) DPPH radical scavenging, (ii) linoleic acid oxidation assay, and (iii) oxidative cell death assay	[93]
